# Reply to Trudzinski et al. Comment on “Akil et al. Nonintubated versus Intubated Lung Volume Reduction Surgery in Patients with End-Stage Lung Emphysema and Hypercapnia. *J. Clin. Med.* 2023, *12*, 3750”

**DOI:** 10.3390/jcm14124161

**Published:** 2025-06-12

**Authors:** Ali Akil, Stephanie Rehers, Stephan Ziegeler, Erik Ernst, Jan Haselmann, Nicolas Johannes Dickgreber, Stefan Fischer

**Affiliations:** 1 Department of Thoracic Surgery, Emden General Hospital, 26721 Emden, Germany; dr.ali.akil.11@gmail.com; 2 Department of Anesthesiology, Intensive Care Medicine and Pain Management, Ibbenbueren General Hospital, 49477 Ibbenbueren, Germany; stephanie.rehers@t-online.de (S.R.); sziegeler@hotmail.com (S.Z.); 3 Department of Respiratory Medicine and Pulmonary Rehabilitation, Karl-Hansen-Hospital, 33175 Bad Lippspringe, Germany; ernst.erik@web.de; 4 Department of Thoracic Surgery and Lung Support, Ibbenbueren General Hospital, 49477 Ibbenbueren, Germany; j-haselmann@web.de; 5 Department of Respiratory Medicine and Thoracic Oncology, Ibbenbueren General Hospital, 49477 Ibbenbueren, Germany; n.dickgreber@mathias-spital.de

In response to the comment letter by Trudzinski et al., we would like to thank the discussants for their interest in our work and their critical discussion [1].

After reporting our initial experience with LVR (lung volume reduction) surgery in spontaneously breathing patients (non-intubated) during surgery with single-site, low-flow veno-venous extracorporeal lung support, we have not further utilized this approach at our institution. After gaining more expertise in the field of non-intubated thoracoscopic surgery, we are now planning to adopt this intriguing strategy in LVR surgery as a careful, elegant and less invasive option with no lung support. However, how to decide whether a patient is able to tolerate a pneumothorax situation during NI-VATS (non-intubated video-assisted thoracoscopic surgery) given underlying end-stage emphysema remains currently unclear, and no data are available. Therefore, all LVRS procedures at our institution are now performed conventionally under general anesthesia with double-lumen tube intubation and no extracorporeal support.

Since our publication, however, we have completely changed the evaluation and treatment process of our lung emphysema patients. Our interdisciplinary program has become a member of the German Emphysema Registry, and according to the requirements of this registry, we have established an interdisciplinary COPD/lung emphysema board, which is led by a pulmonologist in the presence of a specially certified pulmonary radiologist and a thoracic surgeon. In a systematic step-by-step pathway, it is first evaluated whether LVR, in general, can be reasonably offered to the patient or whether the requirements are not fulfilled. If the criteria are fulfilled, all patients are screened for an endoscopic/interventional approach such as endobronchial valve application, which is currently used at our institution, in order to choose the least invasive treatment according to national and international recommendations. Only if such an approach is not reasonably applicable will primary LVR surgery be considered and discussed. All board decisions are documented according to the requirements of the German Emphysema Registry. If surgery is also not applicable, patients are retransferred and further managed by their pulmonologist.

Our program has a strong history of and corresponding experience with LVR surgery, whereas our interventional LVR program is still developing. Therefore, LVR patients at higher risk are currently more likely to be considered for surgery, since we were able to report excellent outcomes and safety in this cohort. However, given the increasing evidence from studies, as stated by the discussants, of promising outcomes after interventional LVR in patients at higher risk, we will likely adopt this into our program in the future as our interventional program and our expertise develops. We strongly believe that interventional LVR will increasingly be the standard treatment of choice for the majority of patients with severe lung emphysema and thoracic hyperinflation, and even more when endoscopic treatment modalities have been developed and tested that allow for ignor collateral ventilation and/or fissure integrity. Although minimally invasive LVR surgery has been shown to be effective and safe, the least invasive and safest procedure for the patient should always be considered. Surgery will likely play a relevant role for troubleshooting or LVR completion after endoscopic treatment, and serve as a primary treatment option for the minority of patients if endoscopic procedures are not applicable.

Once again, we would like to thank the discussants for their comments and their fruitful discussion, which we believe will help to further improve our interdisciplinary LVR program, and we very much look forward to closer collaboration and discussion with leading programs in the field of LVR.
